# Supplementary material for: The Peptidisc, a simple method for stabilizing membrane proteins in detergent-free solution
Source: eLife. 2018 Aug 15;7:e34085. doi: 10.7554/eLife.34085 (PMC6093710; doi:10.7554/eLife.34085)
Supplement: Supplementary file 1. — The length of the scaffold proteins was calculated by multiplying the number of amino acids (aa) by 1.5 Å, which is the rise given by an amino acid structured in an alpha-helix. For the MSPs scaffolds, the number of amino acids was from the TEV cleavage site (ENYLFQ//GXXX) to the C-terminus of the proteins. [file elife-34085-supp1.docx]

| Scaffold | Primary Sequence (N-ter to C-ter) | Length | |
| --- | --- | --- | --- |
|  |  | aa | Å |
| NSP_r_ | FAEKFKEAVKDYFAKFWDPAAEKLKEAVKDYFAKLWD | 37 | 55.5 |
| NSP | DWLKAFYDKVAEKLKEAAPDWFKAFYDKVAEKFKEAF | 37 | 55.5 |
| NSP_rbio_ | Biotin-FAEKFKEAVKDYFAKFWDPAAEKLKEAVKDYFAKLWD | 37 | 55.5 |
| MSP_L156_ | GHHHHHHHDYDIPTTENLYFQ//GSTFSKLREQLGPVTQ  EFWDNLEKETEGLRQEMSKDLEEVKAKVQPYLDDFQK  KWQEEMELYRQKVEPLRAELQEGARQKLHELQEKLSP  LGEEMRDRARAHVDALRTHLAPYSDELRQRLAARLEA  LKENGGAR | 135 | 203 |
| MSP1D1 | GHHHHHHHDYDIPTTENLYFQ//GSTFSKLREQLGPVTQE  FWDNLEKETEGLRQEMSKDLEEVKAKVQPYLDDFQKKW  QEEMELYRQKVEPLRAELQEGARQKLHELQEKLSPLGEE  MRDRARAHVDALRTHLAPYSDELRQRLAARLEALKENGG  ARLAEYHAKATEHLSTLSEKAKPALEDLRQGLLPVLESFKV  SFLSALEEYTKKLNTQ | 190 | 285 |
| MSP1D1E3 | MGHHHHHHHDYDIPTTENLYFQ//GSTFSKLREQLGPVTQE  FWDNLEKETEGLRQEMSKDLEEVKAKVQPYLDDFQKKW  QEEMELYRQKVEPLRAELQEGARQKLHELQEKLSPLGEEM  RDRARAHVDALRTHLAPYLDDFQKKWQEEMELYRQKVEP  LRAELQEGARQKLHELQEKLSPLGEEMRDRARAHVDALRT  HLAPYSDELRQRLAARLEALKENGGARLAEYHAKATEHLST  LSEKAKPALEDLRQGLLPVLESFKVSFLSALEEYTKKLNTQ | 256 | 384 |
