# Supplementary material for: The Peptidisc, a simple method for stabilizing membrane proteins in detergent-free solution
Source: eLife. 2018 Aug 15;7:e34085. doi: 10.7554/eLife.34085 (PMC6093710; doi:10.7554/eLife.34085)
Supplement: Supplementary file 2. [file elife-34085-supp2.docx]

| Reagent | 4% | 12% |
| --- | --- | --- |
| Acrylamide (40%) | 4.9 mL | 14.6 mL |
| Bis-Acrylamide (2%) | 2.7 mL | 8 mL |
| 1.5M Tris-pH 8.8 | 12.5 mL | 12.5 mL |
| Glycerol (10%) | 0 mL | 10 mL |
| dH_2_O | Up to 50 mL | Up to 50 mL |
| APS (10%) | 145 µL | 58 µL |
| TEMED | 14.5 µL | 5.8 µL |
