# Supplementary material for: The Peptidisc, a simple method for stabilizing membrane proteins in detergent-free solution
Source: eLife. 2018 Aug 15;7:e34085. doi: 10.7554/eLife.34085 (PMC6093710; doi:10.7554/eLife.34085)
Supplement: Supplementary file 3. [file elife-34085-supp3.docx]

| **Complex** | | **MalFGK_2_** | | **FhuA/TonB/ColM** | | **SecYEG** | | **BRC** | | **OmpF_3_** | |
| --- | --- | --- | --- | --- | --- | --- | --- | --- | --- | --- | --- |
| Subunit | Mw (kDa) | MalF | 57.0 | FhuA_his_ | 79.7 | SecE_his_ | 14.5 | PufM | 34.5 | OmpF | 37.1 |
|  | | MalK_his_ | 41.9 | TonB_his_ | 23.5 | SecY | 48.5 | PufL | 31.5 |  |  |
|  | | MalG | 32.5 | ColM_his_ | 58.9 | SecG | 11.4 | PuhA | 28.0 |  |  |
